# Assessing Anthocyanin Biosynthesis in Solanaceae as a Model Pathway for Secondary Metabolism

**DOI:** 10.3390/genes10080559

**Published:** 2019-07-25

**Authors:** Zuo Li, Trisha L. Vickrey, Moira G. McNally, Shirley J. Sato, Tom Elmo Clemente, Jeffrey P. Mower

**Affiliations:** 1 Center for Plant Science Innovation, University of Nebraska, Lincoln, NE 68588, USA; 2 Guangdong Key Laboratory of Ornamental Plant Germplasm Innovation and Utilization, Environmental Horticulture Research Institute, Guangdong Academy of Agricultural Sciences, Guangzhou 510640, China; 3 Department of Chemistry, University of Nebraska, Lincoln, NE 68588, USA; 4 Biology Department, University of Jamestown, Jamestown, ND 58405, USA; 5 Center for Biotechnology, University of Nebraska, Lincoln, NE 68588, USA; 6 Department of Agronomy and Horticulture, University of Nebraska, Lincoln, NE 68583, USA

**Keywords:** anthocyanin biosynthesis, comparative genomics, reverse genetics, Solanaceae

## Abstract

Solanaceae have played an important role in elucidating how flower color is specified by the flavonoid biosynthesis pathway (FBP), which produces anthocyanins and other secondary metabolites. With well-established reverse genetics tools and rich genomic resources, Solanaceae provide a robust framework to examine the diversification of this well-studied pathway over short evolutionary timescales and to evaluate the predictability of genetic perturbation on pathway flux. Genomes of eight Solanaceae species, nine related asterids, and four rosids were mined to evaluate variation in copy number of the suite of FBP enzymes involved in anthocyanin biosynthesis. Comparison of annotation sources indicated that the NCBI annotation pipeline generated more and longer FBP annotations on average than genome-specific annotation pipelines. The pattern of diversification of each enzyme among asterids was assessed by phylogenetic analysis, showing that the CHS superfamily encompasses a large paralogous family of ancient and recent duplicates, whereas other FBP enzymes have diversified via recent duplications in particular lineages. Heterologous expression of a pansy F3′5′H gene in tobacco changed flower color from pink to dark purple, demonstrating that anthocyanin production can be predictably modified using reverse genetics. These results suggest that the Solanaceae FBP could be an ideal system to model genotype-to-phenotype interactions for secondary metabolism.

## 1. Introduction

In many plants, anthocyanins are the major secondary metabolites that determine fruit and flower color, which is important for plant reproduction by attracting agents for pollination and seed dispersal [1]. The production of anthocyanidins, the immediate precursors of anthocyanins, involves a series of enzymatic reactions in the flavonoid biosynthesis pathway (FBP). Chalcone synthase (CHS), chalcone isomerase (CHI), and flavanone 3-hydroxylase (F3H) are early pathway enzymes that commit to the production of flavonoids. Flavonoid 3′-monooxygenase (F3′H) and flavonoid 3′,5′-hydroxylase (F3′5′H) direct flux to a particular branch of the pathway to produce different anthocyanidins with distinct color profiles, and dihydroflavonol 4-reductase (DFR) and anthocyanidin synthase (ANS) make additional modifications to generate three major anthocyanidins (cyanidin, delphinidin, pelargonidin), from which three additional anthocyanidins (malvidin, peonidin, petunidin) can be derived by methylation. Expression of FBP enzymes is regulated by a suite of transcription factors in the R2R3-MYB, bHLH, and WDR multigene families [2,3]. In addition to anthocyanins, the FBP produces an array of other plant secondary compounds (e.g., flavones, flavonols, isoflavonoids, lignins, and tannins) that participate in plant ecological and physiological functions, such as stress protection, auxin transport and nodulation signaling [1,4,5,6].

Solanaceae (the nightshade family) are an emerging powerhouse for plant evolutionary and comparative genomics. This family comprises 90–100 genera and 2500–3000 species [7,8], including many economically important plants such as tobacco (*Nicotiana tabacum*), potato (*Solanum tuberosum*), tomato (*S. lycopersicum*), petunia (*Petunia hybrida*), sweet and spicy peppers (*Capsicum* spp.), and eggplant (*S. melongena*). To date, over a dozen draft genomes are available from these and other major solanaceous crops as well as many of their progenitors and wild relatives [9,10,11,12,13,14,15,16,17,18,19,20,21]. In addition, genomes from successively more distantly related species of asterids are available, providing broader evolutionary context for comparative analyses. The morning glory (*Ipomoea nil* [22]) genome represents Convolvulaceae, the sister family to Solanaceae in Solanales. Lamiales and Gentianales, the two orders closest to Solanales, are represented by genomes from monkeyflower (*Mimulus guttatus* [23]), sesame (*Sesamum indicum* [24]), robusta coffee (*Coffea canephora* [25]), and olive (*Olea europaea* [26,27]). Sister to the lamiids, to which all aforementioned plants belong, are the campanulids, with genomes available for carrot (*Daucus carota* [28]), sunflower (*Helianthus annuus* [29]), lettuce (*Lactuca sativa* [30]), and wild artichoke (*Cynara cardunculus* [31]).

With such diverse availability of genomes from a single family, Solanaceae provide an ideal platform to perform comprehensive studies on the evolutionary diversification of pathways such as the FBP. Indeed, many Solanaceae species exhibit diversity in the types and relative levels of different anthocyanins in their flowers and fruits: pink tobacco flowers primarily contain cyanidin [32], the dark purple color of eggplant skins and *Iochroma* flowers is produced by delphinidin [33,34], many red-fleshed potato cultivars derive their coloration from pelargonidin [35,36], and all six of the major anthocyanidins can be variously found in different potato cultivars [37]. Moreover, petunia and tobacco have played key roles in the elucidation of FBP enzyme function due to the early availability of efficient plant transformation systems for these species. Transgenic tobacco and petunia lines have been used to demonstrate increased anthocyanin biosynthesis by introduction or overexpression of *DFR*, *F3′H*, or *F3′5′H* [38,39,40,41], reduced anthocyanin biosynthesis by antisense RNA or RNAi suppression of *CHS* or *CHI* [42,43,44], and altered FBP flux after introduction or suppression of FBP enzymes that catalyze the production of non-anthocyanin products such as flavonols, flavones, isoflavones, and stilbenoids [41,45,46,47,48].

Here, we used the extensive genomic and reverse genetic resources for Solanaceae and related species to advance anthocyanin biosynthesis in Solanaceae as a model pathway for secondary metabolism. We conducted a comprehensive survey on the diversity of FBP enzymes involved in anthocyanin biosynthesis to establish the variation in presence and copy-number of anthocyanin biosynthesis genes among Solanaceae and other eudicots. Because gene copy number is dependent on the quality of gene annotation, we also assessed the consistency of FBP gene annotation between different annotation sources for most species. Phylogenetic analysis was used to identify the pattern of FBP enzyme diversification via gene duplication. Finally, a useful system for pathway modeling requires that simple pathway perturbations should result in predictable changes in phenotype. Thus, we transgenically introduced pansy *F3′5′H* into tobacco to demonstrate the reliability with which flower color can be predictably altered via genetic modification. These results contribute to the extensive body of literature on the evolution and genetic manipulation of the FBP pathway in Solanaceae, and sets up the Solanaceae FBP as a potentially powerful system to model genotype-to-phenotype interactions in secondary metabolism.

## 2. Materials and Methods 

### 2.1. Identification of Flavonoid Biosynthesis Pathway Homologs

Gene sequences from six representative eudicots (*Arabidopsis thaliana*, *D. carota*, *M. guttatus*, *S. lycopersicum*, *S. tuberosum*, and *Vitis vinifera*) were downloaded from Phytozome using KEGG codes for CHS (KEGG code K00660), CHI (K01859), F3H (K00475), F3′H (K05280), F3′5′H (K13083), DFR (K13082), and ANS (K05277). These sequences were used as queries in relaxed blastn searches (-task blastn -penalty −1 -reward 1 -evalue 1e^−50^) to find coding sequence homologs in 39 independent genome annotations from 21 species of eudicots, with a focus on solanaceous crops. Annotated coding sequences created by the NCBI Eukaryotic Genome Annotation Pipeline were obtained for 18 species, while annotated coding sequences created by genome-specific pipelines were obtained from Phytozome version 12.1 for 10 species, from the Sol Genomics Network for 8 species, and from genome-specific websites for 3 species (Table S1). In total, two independent sets of annotated coding sequences were obtained for 18 species, while a single annotation was obtained for 3 species.

When homologous sequences included 5′ or 3′ UTRs, the program TransDecoder v5.3 [49] was used to infer the coding sequence, requiring a minimum coding sequence length of 300 bp. For each species, transcript variants arising from a single locus were eliminated by several approaches, depending on source of the annotated gene sequences. For annotations in Phytozome, the ‘primary transcripts only’ data sets were used, which are prefiltered to contain a single transcript per locus. For NCBI pipeline annotations, transcript variants were excluded by keeping the longest transcript sequence for every defined locus number. For data downloaded from the Sol Genomics Network and species-specific websites, generic feature format annotation files were consulted to keep only the single longest annotated transcript for each locus.

### 2.2. Comparison of Annotations from Different Sources

To assess the level of agreement between annotations from different sources, annotations from the NCBI Eukaryotic Genome Annotation Pipeline were compared with annotations from genome-specific pipelines (available from Phytozome, Sol Genomics Network, and genomic-specific websites) for 18 species (Table S1). Using blastn with default values, the annotated genes from each source were classified as perfect pairs, imperfect pairs, or unpaired. ‘Perfect pairs’ were reciprocal-best hits (RBHs) with 100% identity over 100% of the length of the query sequence in a default blastn search. ‘Imperfect pairs’ were RBHs with <100% identity and/or different length relative to the query sequence. Annotations that did not have a perfect or imperfect pair between sources were initially classified as ‘unpaired’. Unpaired annotations were excluded if their coding sequences were identical to another annotated gene from the same annotation source.

### 2.3. Phylogenetic Analysis of Flavonoid Biosynthesis Enzymes

Gene annotations from both annotation sources were examined phylogenetically to determine whether any unpaired genes clustered in a phylogenetic tree. DNA sequences for each flavonoid biosynthesis enzyme were aligned with Muscle v3.8.31 [50] using default parameters. Aligned enzyme sequences were trimmed using Gblocks v0.91b [51] in codon mode, with the minimum block length parameter (b4) set to 5 and the parameters for minimum flanking position sequence (b2) and gaps allowed (b5) set to half the total number of aligned sequences. Phylogenetic trees were constructed from each of the trimmed sequence alignments using the maximum likelihood implementation in RAxML v8.2.4 [52]. For each enzyme data set, the general time reversible model with a gamma distribution of rate variation was used. During the analysis, the shape of the gamma distribution and substitution rate parameters were estimated, and a fast bootstrap analysis with 1000 replicates was performed to assess support for branches. Unpaired genes from different annotation sources that formed a unique cluster in the phylogenetic results were reclassified as ‘imperfect pairs’.

Final data sets were created for each FBP gene by keeping only one annotation of the perfect and imperfect pairs, and in these cases, the NCBI pipeline annotations were kept over the genome-specific pipeline annotations for consistency. These final data sets for each FBP gene were aligned using Muscle, trimmed using Gblocks, and phylogenetically analyzed using RAxML as described above. Because the CHS data set comprises a family of paralogous genes, the resulting tree was rooted using non-asterid species in order to evaluate the relationship among asterid CHS paralogs. All other genes were unrooted analyses of asterid sequences.

### 2.4. Evaluation of Flower Color Modification Using Transgenic F3′5′H

The *F3′5′H* gene from *Viola* × *wittrockiana*, which was previously shown to modify flower color from pink/red hues to blue/purple hues in several plant species [53,54], was chosen for transgene analysis in this study. An expression cassette was synthesized (GenScript Corp., Piscataway, NJ, USA) containing the complete *V. wittrockiana F3′5′H* coding sequence (GenBank accession number AB332097) coupled with a translational enhancer from tobacco etch virus, which were situated between an enhanced 35S promoter from cauliflower mosaic virus and a polyadenylation signal. This expression cassette was subcloned into the binary vector pPZP212, which contains the selectable marker *NptII* [55], and introduced into *Agrobacterium tumefaciens* C58C1/pMP90 by triparental mating [56]. Transformation was carried out using tobacco (*N. tabacum* cv. Xanthi) leaf discs according to established protocols [57]. Regenerated plants (T_0_ generation) and seeds collected from self-pollinated T_0_ plants (T_1_ generation) or wild type Xanthi plants (WT) were grown to maturity in a glasshouse, and flower color was assessed by eye.

Expression of *NptII* was monitored in selected T_0_ and T_1_ plants by an NPTII ELISA kit (Agdia Inc., Elkhart, IN, USA) according to the supplied protocol. The presence and expression of the *F3′5′H* transgene was evaluated in selected T_0_ and T_1_ plants through polymerase chain reaction (PCR) and reverse transcription PCR (RT-PCR), respectively, using the native *RPL23A* gene as a standard control. To assess the presence of the transgene, genomic DNA was extracted from a mature leaf according to a CTAB procedure [58], and PCR was performed with 20 ng of DNA using gene-specific primers for *F3′5′H* (CCGAGTCTAACGAGTTCAAAG; CCCATTTGTATTGTCGCATTC) and *RPL23A* (GCACCTGGAAGGAACAAA; ACGTCCAAAGCATCATAGTC) according to a previously described PCR protocol [59]. Cellular RNA was extracted from a mature flower and 2 µg was converted to cDNA according to established procedures [60]. Expression of *F3′5′H* and *RPL23A* was assayed by RT-PCR using a semi-quantitative approach in which reactions were amplified for 20, 24, 28, or 32 cycles of denaturation (95 °C for 30 s), annealing (55 °C for 30 s), and elongation (72 °C for 90 s).

To compare anthocyanin content of T_1_ flowers to WT, the corolla lobe from individual flowers were collected upon full flower opening and then stored at −80 °C. Anthocyanins were eluted overnight in 1 mL of acidified methanol with 1% HCl. Absorbance was measured at 530 nm using a GENESYS 150 UV-Vis spectrophotometer for 3–4 flowers from each of four T_1_ transgenic lines and WT. Absorbance values from transgenic lines were compared to WT plants using an unpaired *t*-test. For thin-layer chromatography analysis, silica gel plates (10 × 20 cm, Merck) were used as a stationary phase and n-butanol:acetic acid:water (4:1:5, v/v/v, upper phase) was used as a mobile phase. Approximately 2 µL of sample was spotted onto the plate by adding a small amount at a time using a 10 µL-pipette. Samples were spaced 1 cm apart. Plates were developed in a tank with the solvent mixture. After developing, the plates were dried and pigments visualized directly.

## 3. Results

### 3.1. The NCBI Pipeline Annotates More and Longer Anthocyanin Biosynthesis Enzymes

**Figure 1:**
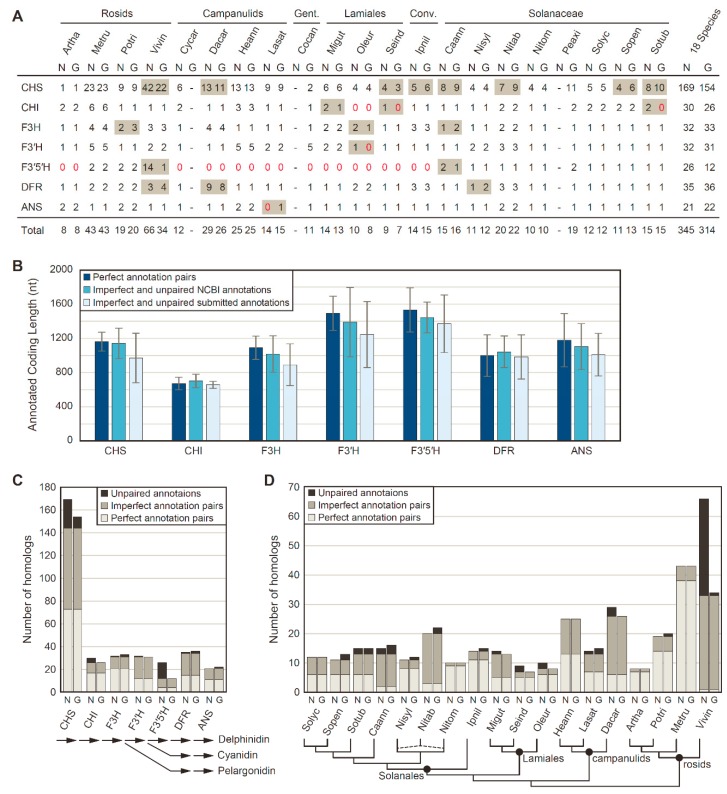
Homology detection of anthocyanin biosynthesis genes between NCBI (N) and genome-specific (G) pipeline annotations. (**A**) Anthocyanin biosynthesis gene counts. Discrepant values between annotation sources are shaded in gray. Zero values are denoted with red text. The sum totals at right include only the 18 species with NCBI and genome-specific pipeline annotations. Conv., Convolvulaceae; Gent., Gentianales. (**B**) Mean ± SD for coding sequence lengths in nucleotides (nt) of anthocyanin biosynthesis genes. (**C**) Number of homologs identified for each anthocyanin biosynthesis gene. A simplified anthocyanin biosynthesis pathway is shown at the bottom. (**D**) Number of homologs identified for each species. Phylogenetic relationships among sampled taxa is provided at the bottom. The dashed lines for *N. tabacum* (Nitab) indicates its allopolyploid nature, with S genome and T genome contributions from *N. sylvestris* (Nisyl) and *N. tomentosum* (Nitom), respectively.

Putative homologs to seven FBP enzymes (CHS, CHI, F3H, F3′H, F3′5′H, DFR, and ANS) were identified in each of 39 genome annotations from 21 different eudicots by blast homology searches, followed by filtration to remove short coding sequences (<300 bp) and transcript variants (Figure 1A). To assess annotation consistency between NCBI pipeline annotations and genome-specific pipeline annotations stored in various data repositories, blast searches and phylogenetic clustering were used to categorize each FBP homolog as a perfect pair, an imperfect pair, or an unpaired sequence for the 18 species with both annotation types. For these 18 species, 345 FBP homologs were identified in the NCBI-based pipeline annotations, whereas 314 FBP homologs were identified in the genome-specific pipeline annotations. Of these, 299 homologs were shared between both annotation sets, with identical coding sequence annotations for about half of the shared homologs (153 perfect pairwise matches; 146 imperfect pairwise matches). However, 46 NCBI pipeline annotations and 15 genome-specific pipeline annotations did not have a pairwise match in the other annotation set. In terms of length, the imperfect pairs and unpaired annotations were longer for all enzymes on average from the NCBI pipeline annotation compared with genome-specific pipeline annotations (Figure 1B).

The number of detected homologs varied substantially among FBP enzymes (Figure 1C) and species (Figure 1D). Notably, substantially more homologs were recovered per species for *CHS* compared with other enzymes, due to the fact that *CHS* is a member of a larger chalcone synthase superfamily that includes related proteins with non-CHS functions [61,62]. Thus, it is likely that only a fraction of the *CHS* homologs detected in this analysis encode true CHS proteins that synthesize chalcones, and hereafter, we will refer to the detected *CHS* homologs as the *CHS* family. Overall, the NCBI and genome-specific pipeline annotations were largely in agreement in terms of the number of homologs per gene and per species, as demonstrated by the majority of homologs having a perfect or imperfect pair between annotation sources, although relatively large discrepancies between annotation results were detected for the *CHS* family, for *F3′5′H*, and for *V. vinifera* (Figure 1C; Figure 1D). These discrepancies were primarily caused by the recent lineage-specific expansions of these two gene families in *V. vinifera* [63,64], with more of these recent duplicates detected in the *V. vinifera* annotation produced by NCBI than in the *V. vinifera* annotation obtained from Phytozome (Figure 1A).

### 3.2. Rampant Lineage-Specific Duplication of Anthocyanin Biosynthesis Genes in Asterids

**Figure 2:**
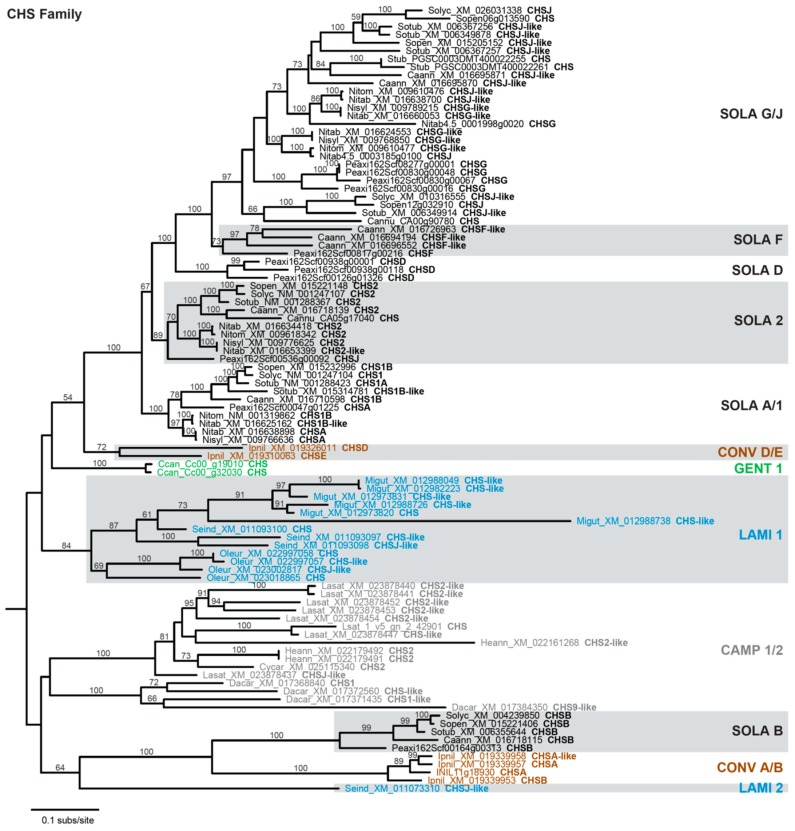
Phylogram depicting phylogenetic relationships among *CHS* family homologs. Taxon names include a species abbreviation, a GenBank accession number or gene ID, and the Chalcone synthase (CHS) subfamily designation taken from the accession. Bootstrap values >50% are shown. Monophyletic clades are labeled as described in text and color-coded according to phylogenetic grouping: Campanulids, gray; Convolvulaceae, brown; Gentianales, green; Lamiales, blue; Solanaceae, black. Non-asterid sequences used to root the tree are not shown.

The results from Figure 1 corroborate that *CHS* has many paralogs in most species and indicate some level of gene duplication for the other FBP enzymes. Phylogenetic analysis was performed to examine the timing of duplication and relationship among these paralogs. Of the seven anthocyanin biosynthesis genes, the *CHS* family exhibits the most dynamic evolutionary history, with multiple paralogous gene groups for most sampled asterids (Figure 2). Six paralogous groups were recovered for Solanaceae, corresponding closely with the A+B+D+F+G+J groups of *CHS* homologs previously defined for *Petunia* [65]. Five of the Solanaceae groups are most closely related to one another, implying a complex history of gene duplications and losses during Solanaceae diversification. The sixth Solanaceae group (SOLA B) contained a single copy for *Capsicum*, *Petunia*, and *Solanum*, indicating a recent loss of this homolog from *Nicotiana*. For *Ipomoea* (the sole representative of Convolvulaceae), two distinct paralogous groups (CONV A/B, CONV D/E) were identified that correspond to the previously described A+B+C and D+E groups [66], and these two clades are sister with the two Solanaceae clades. For Lamiales, one paralog group (LAMI 1) had multiple representatives for all three species, while the second group (LAMI 2) was represented by a single gene from *Sesamum*. Only one paralog group (GENT 1) was obtained for *Coffea*, the lone representative of Gentianales, and for campanulids (CAMP 1/2). 

**Figure 3:**
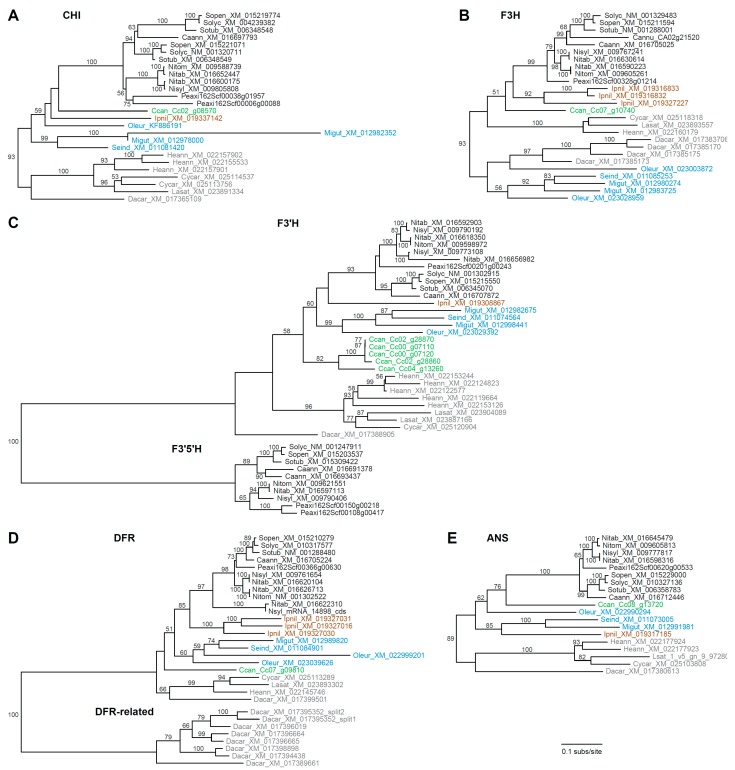
Phylograms depicting phylogenetic relationships among anthocyanin biosynthesis genes. (**A**) *CHI*, (**B**) *F3H*, (**C**) *F3′H* and *F3′5′H*, (**D**) *DFR*, and (**E**) *ANS*. All trees are drawn to the same scale shown at the bottom. Taxon names include a species abbreviation and GenBank accession number or gene ID. Bootstrap values >50% are shown. Major clades are color coded as in Figure 2.

Compared with *CHS*, the remaining FBP genes are less evolutionarily dynamic, with far fewer paralog copies on average, and each tree was largely congruent with organismal phylogeny (Figure 3). For *CHI* (Figure 3A), lineage-specific duplications were detected for *Solanum*, *Petunia*, *Mimulus, Helianthus*, and *Cynara*. The *F3H* tree (Figure 3B) revealed lineage-specific duplications for *Capsicum*, *Ipomoea*, *Daucus*, *Mimulus*, and *Olea*. Unexpectedly, campanulids and lamiids were not recovered as monophyletic groups in the *F3H* tree, which suggests either differential loss of two ancestral paralogs or a phylogenetic artifact. The *F3′H* and *F3′5′H* were evaluated together (Figure 3C) as they are members of the CYP75 subfamily of cytochrome P450 genes [67]. Lineage-specific duplications of *F3′H* were apparent for *Mimulus*, *Coffea*, *Helianthus*, *Lactuca*, *N. sylvestris,* and the S genome of *N. tabacum*, while *F3′5′H* homologs were not identified from any asterids except Solanaceae, with duplications affecting *Petunia* and *Capsicum*. For *DFR* (Figure 3D), three *Ipomoea* homologs resulted from recent tandem duplication events [68], and an additional truncated gene was present for *N. sylvestris*, the S genome of *N. tabacum*, and *Olea*. Additionally, a multigene paralogous group was detected for *Daucus* (the *DFR*-related clade). The *Daucus DFR* gene in the species-rich *DFR* clade was orthologous to the previously characterized DcDFR1 [69,70], while the other *DFR*-like paralogs were more similar to DcDFR2, which does not seem to participate in anthocyanin biosynthesis [69]. The ANS tree revealed just a single duplication for *Helianthus* (Figure 3E).

### 3.3. Overexpression of F3′5′H Produces Purple Flowers in Tobacco cv. Xanthi

The *N. tabacum* genome encodes all seven FBP enzymes for anthocyanin biosynthesis, yet the flowers of most cultivars are pink due to the predominance of cyanidin, as well as its metabolic precursors dihydrokaempferol (DHK) and dihydroquercetin (DHQ), in petals [32]. Because DHK and DHQ are present, it was hypothesized that FBP flux could be altered from cyanidin to delphinidin via introduction of a highly expressed *F3′5′H* gene that produces dihydromyricetin (DHM), the precursor of delphinidin, from DHK and DHQ. Thus, we transformed tobacco cv. Xanthi with a construct containing a pansy *F3′5′H* transgene under control of an enhanced 35S promoter. Eighteen regenerated plants (T_0_) were grown to maturity, with flower colors ranging from WT light pink to dark purple. Expression of *NptII* was confirmed for 12 of 18 plants, of which 7 plants exhibited a solid or striped purple flower phenotype. 

**Figure 4:**
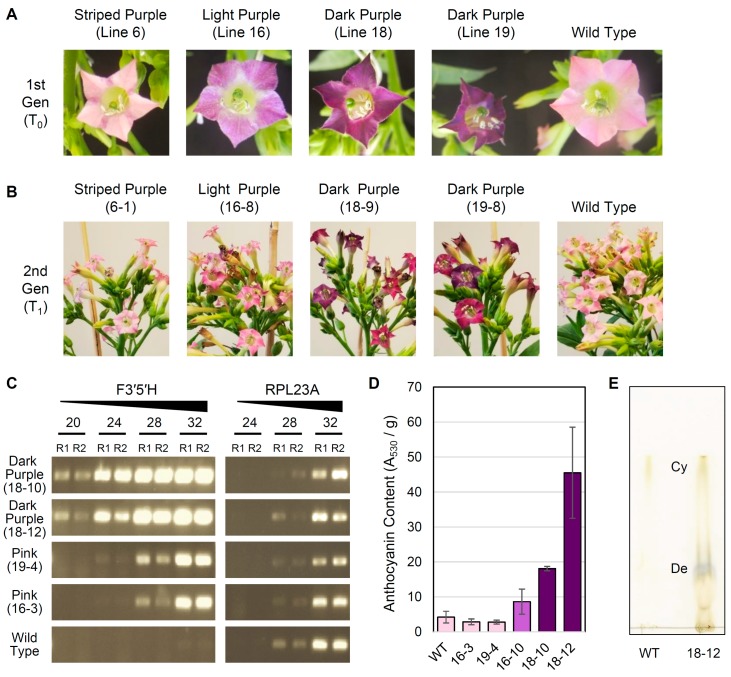
Characterization of transgenic tobacco lines. (**A**) Representative flowers from the T_0_ generation and wild type tobacco. (**B**) Representative inflorescences from the T_1_ generation and wild type tobacco. (**C**) Semi-quantitative reverse transcription PCR (RT-PCR) analysis of transgenic *F3′5′H* and native *RPL23A* expression in T_1_ individuals and wild type tobacco. RT-PCR reactions were stopped at 20, 24, 28, and 32 PCR cycles and run on a 1.5% agarose gel, and reactions were performed in replicate (R1, R2). (**D**) Mean ± 2×SEM for anthocyanin content (measured as absorbance at 530 nm per g tissue) in stage 12 flowers from T_1_ individuals and wild type (WT) tobacco. Histogram bars for each sampled individual are color coded according to approximate flower color. (**E**) Thin-layer chromatography comparing cyanidin (Cy) and delphinidin (De) content in flowers from line 18-12 and WT.

To assess the stability of transformation, seeds from self-pollinated T_0_ plants were collected from four lines (Figure 4A) exhibiting a variety of mutant flower color phenotypes, including striped purple (L6), light purple (L16), and dark purple (L18, L19). For each T_0_ line, a population of twelve T_1_ plants were grown to maturity, and their flower colors were evaluated (Figure 4B; Figure S1). For L18, all T_1_ plants had dark purple flowers. For L19, most T_1_ plants exhibited either dark or light purple flowers, but three T_1_ plants had pink flowers similar to WT. For L16, most T_1_ plants had light purple flowers, except that two T_1_ plants had pink flowers like WT. For L6, one T_1_ plant had dark purple flowers while the remaining T_1_ plants either exhibited inconsistent purple striping (present in only some flowers) or pink flowers consistent with WT.

As expected, the relative expression level of the *F3′5′H* transgene was higher in T_1_ plants with dark purple flowers than those with pink flowers (Figure 4C), which was indicated by the much earlier appearance of RT-PCR products starting at cycle 20 for the dark purple lines compared with cycle 24 or 28 for the light purple lines, even though the same amount of starting cDNA material was used for each reaction. No major variation was observed in the relative expression level of *RPL23A*, confirming that similar amounts of cDNA were present in each reaction, and no expression of the *F3′5′H* transgene was detected in WT plants. Additionally, relative anthocyanin content (measured by absorbance at 530 nm per g of tissue) correlated well with the intensity of purple coloration in representative T_1_ individuals (Figure 4D). Dark purple flowers from two different T_1_ plants (18-10 and 18-12) had significantly higher anthocyanin content than WT flowers (unpaired *t*-test, *P* = 0.0001 and *P* = 0.003, respectively). Anthocyanin content was also 2-fold higher in light purple flowers from T_1_ line 16-10 compared with WT, although this difference was not significant (*P* = 0.12). For two T_1_ lines (16-3 and 19-4) with light pink flowers comparable to WT, floral anthocyanin content was slightly lower but not significantly different from WT (*P* = 0.13 and *P* = 0.22). Finally, thin-layer chromatography (Figure 4E) indicated that the dark purple flowers in line 18-12 were the result of a shift in anthocyanin production to primarily delphinidin, with a minor but detectable amount of cyanidin also produced. In contrast, WT tobacco cv. Xanthi contained detectable levels of cyanidin only, as expected [32].

## 4. Discussion and Conclusions

In this study, we examined the presence and copy number of seven FBP enzymes involved in anthocyanin biosynthesis from 21 solanaceous and related eudicot species. One goal of this study was to compare the consistency of annotations from different sources, such as the NCBI Eukaryotic Genome Annotation Pipeline relative to genome-specific pipeline annotations hosted at Phytozome, the Sol Genomics Network, and genome-specific websites (Table S1). Overall, the large majority of FBP homologs detected from these various annotation sources were in close agreement, but when they differed, homologs from the NCBI annotations were generally longer and more abundant than from the genome-specific databases (Figure 1). These results suggest that reannotation of genome sequences using a unified annotation strategy, such as employed by the NCBI pipeline, may be preferable for improved consistency during comparative genomics research.

A second goal of this research was to assess the diversity of gene content for anthocyanin biosynthesis enzymes among sequenced asterid genomes. In particular, we were interested in assessing whether homology assessment might provide a reliable indicator of gene function. For *CHS*, homology assessment recovered a large gene family exhibiting extensive gene duplication (Figure 2). Neofunctionalization of *CHS* duplicates is widespread [61,71], with particular examples of independent functional transitions from chalcone to non-chalcone production in Asteraceae and *Vitis* [63,72,73]. Subfunctionalization has also been demonstrated for *CHS*; of the multiple *CHS* genes in petunia and *Ipomoea*, only one or two are expressed in flower petals [66,74], while in common bean, several *CHS* homologs are differentially expressed in response to various environmental stressors [75]. Thus, for *CHS*, homology assessment is insufficient to identify the most likely candidates involved in anthocyanin biosynthesis, and molecular characterization is needed to assess protein localization and function. For the other six enzymes, however, phylogenetic analysis of identified homologs produced trees that generally recapitulated organismal relationships, with occasional duplications in particular species (Figure 3). This suggests that many of these genes likely maintain an important role in anthocyanin biosynthesis, with the potential for subfunctionalization in species that contain multiple recent duplicates.

A final goal of this research was to explore the possibility of advancing the Solanaceae FBP as a quantitative model for genotype-to-phenotype interactions of secondary metabolism. One of the most important goals of biological research in the postgenomics era is to establish how genotype determines phenotype. Flower color provides an ideal system to examine this relationship because the genotype (the flavonoid biosynthesis pathway, which produces anthocyanin pigments) is genetically well described, the phenotype (flower color) is directly observable, and the metabolic intermediates (e.g., chalcone, DHK, DHQ, DHM, pelargonidin, cyanidin, and delphinidin) are well known. To be a useful model, simple perturbations should result in predictable phenotypic shifts. In tobacco, the presence of anthocyanin precursors DHK and DHQ suggested that flux could be altered by transgenic overexpression of *F3′5′H*, which converts DHK and DHQ to DHM and commits the pathway to delphinidin biosynthesis. Indeed, several transgenic tobacco lines exhibited clear shifts in flower color from light pink to various purple phenotypes (Figure 4), consistent with previous observations [39,40]. 

More generally, there are many studies demonstrating flower color evolution resulting from perturbations of the anthocyanin pathway due to functional gene loss [76,77] or to changes in enzyme sequence [78,79] or gene expression levels [80,81,82]. Several studies have begun to take advantage of the FBP as a quantitative model by evaluating gene expression and metabolites [83,84,85,86,87], yet fine-scale transgenic manipulation of the pathway and quantitative hypothesis testing of such changes on pathway flux are now possible, particularly in Solanaceae where so many genomes and reverse genetic systems are available. Ultimately, development of the Solanaceae FBP as a manipulable model for secondary metabolism could be used to quantitatively track the effects of complex perturbations on relative expression of enzymes and transcription factors and relative abundance of metabolic intermediates and anthocyanins. 

## Supplementary Materials

The following are available online at https://www.mdpi.com/2073-4425/10/8/559/s1, Figure S1: Populations of T1 generation lines in comparison to WT, Table S1: Sources of coding sequence annotations, Table S2: Comparison of anthocyanin biosynthesis genes from NCBI and genome-specific annotations.
