# Supplementary material for: Assessing Anthocyanin Biosynthesis in Solanaceae as a Model Pathway for Secondary Metabolism
Source: Genes (Basel). 2019 Jul 25;10(8):559. doi: 10.3390/genes10080559 (PMC6723469; doi:10.3390/genes10080559)
Supplement: Supplementary file 1 [file genes-10-00559-s001.zip › SuppFile/SuppFigS1TableS1TableS2.pdf]

Table S1. Sources of coding sequence annotations

Group	Order	Species	Abbreviation	Version	Source
lamiids	Gentianales	*Coffea canephora*	Cocan	Ccanephora	solgenomics.net
lamiids	Lamiales	*Mimulus guttatus*	Migut	Mimgu1_0	ncbi.nlm.nih.gov/assembly
lamiids	Lamiales	*Mimulus guttatus*	Migut	v2.0	phytozome.jgi.doe.gov
lamiids	Lamiales	*Olea europaea*	Oleur	O_europaea_v1	ncbi.nlm.nih.gov/assembly
lamiids	Lamiales	*Olea europaea*	Oleur	v1.0	phytozome.jgi.doe.gov
lamiids	Lamiales	*Sesamum indicum*	Seind	S_indicum_v1.0	ncbi.nlm.nih.gov/assembly
lamiids	Lamiales	*Sesamum indicum*	Seind	v1.0	ocri-genomics.org/Sinbase
lamiids	Solanales	*Capsicum annuum*	Caann	Zunla_1_Ref_v1.0	ncbi.nlm.nih.gov/assembly
lamiids	Solanales	*Capsicum annuum*	Caann	CM334 v.1.55	solgenomics.net
lamiids	Solanales	*Ipomoea nil*	Ipnil	Asagao_1.1	ncbi.nlm.nih.gov/assembly
lamiids	Solanales	*Ipomoea nil*	Ipnil	Asagao_1.2	viewer.shigen.info/asagao
lamiids	Solanales	*Nicotiana sylvestris*	Nisyl	Nsyl	ncbi.nlm.nih.gov/assembly
lamiids	Solanales	*Nicotiana sylvestris*	Nisyl	Nsyl_ASAF01	solgenomics.net
lamiids	Solanales	*Nicotiana tabacum*	Nitab	Ntab-TN90	ncbi.nlm.nih.gov/assembly
lamiids	Solanales	*Nicotiana tabacum*	Nitab	v4.5	solgenomics.net
lamiids	Solanales	*Nicotiana tomentosiformis*	Nitom	Ntom_v01	ncbi.nlm.nih.gov/assembly
lamiids	Solanales	*Nicotiana tomentosiformis*	Nitom	Ntom_ASAG01	solgenomics.net
lamiids	Solanales	*Petunia axillaris*	Peaxi	v1.6.2	solgenomics.net
lamiids	Solanales	*Solanum lycopersicum*	Solyc	SL3.0	ncbi.nlm.nih.gov/assembly
lamiids	Solanales	*Solanum lycopersicum*	Solyc	ITAG3.2	solgenomics.net
lamiids	Solanales	*Solanum pennellii*	Sopen	SPENNV200	ncbi.nlm.nih.gov/assembly
lamiids	Solanales	*Solanum pennellii*	Sopen	v2	solgenomics.net
lamiids	Solanales	*Solanum tuberosum*	Sotub	SolTub_3.0	ncbi.nlm.nih.gov/assembly
lamiids	Solanales	*Solanum tuberosum*	Sotub	v4.03	phytozome.jgi.doe.gov
campanulids	Apiales	*Daucus carota*	Dacar	ASM162521v1	ncbi.nlm.nih.gov/assembly
campanulids	Apiales	*Daucus carota*	Dacar	v2.0	phytozome.jgi.doe.gov
campanulids	Asterales	*Cynara cardunculus*	Cycar	CcrdV1	ncbi.nlm.nih.gov/assembly
campanulids	Asterales	*Helianthus annuus*	Heann	HanXRQr1.0	ncbi.nlm.nih.gov/assembly
campanulids	Asterales	*Helianthus annuus*	Heann	r1.2	phytozome.jgi.doe.gov
campanulids	Asterales	*Lactuca sativa*	Lasat	Lsat_Salinas_v7	ncbi.nlm.nih.gov/assembly
campanulids	Asterales	*Lactuca sativa*	Lasat	v5	phytozome.jgi.doe.gov
rosids	Brassicales	*Arabidopsis thaliana*	Artha	TAIR10	ncbi.nlm.nih.gov/assembly
rosids	Brassicales	*Arabidopsis thaliana*	Artha	TAIR10	phytozome.jgi.doe.gov
rosids	Fabales	*Medicago truncatula*	Metru	MedtrA17_4.0	ncbi.nlm.nih.gov/assembly
rosids	Fabales	*Medicago truncatula*	Metru	Mt4.0v1	phytozome.jgi.doe.gov
rosids	Malpighiales	*Populus trichocarpa*	Potri	Pop_tri_v3	ncbi.nlm.nih.gov/assembly
rosids	Malpighiales	*Populus trichocarpa*	Potri	v3.0	phytozome.jgi.doe.gov
rosids	Vitales	*Vitis vinifera*	Vivin	12X	ncbi.nlm.nih.gov/assembly
rosids	Vitales	*Vitis vinifera*	Vivin	12X	phytozome.jgi.doe.gov

Table S2. Comparison of anthocyanin biosynthesis genes from NCBI and genome-specific annotations

		NCBI Annotation		Genome-specific Annotation			
Gene	Species	Gene	Length	Gene	Length	Perc ID	Classification
ANS	Artha	AT4G22870.1	339	**AT4G22870.2**	462	100.0	Imperfect RBH
ANS	Artha	**AT4G22880.1**	1071	AT4G22880.1	1071	100.0	Perfect RBH
CHI	Artha	**AT3G55120.1**	741	AT3G55120.1	741	100.0	Perfect RBH
CHI	Artha	**AT5G66220.1**	525	AT5G66220.1	525	100.0	Perfect RBH
CHS	Artha	**AT5G13930.1**	1188	AT5G13930.1	1188	100.0	Perfect RBH
DFR	Artha	**AT5G42800.1**	1149	AT5G42800.1	1149	100.0	Perfect RBH
F3′H	Artha	**AT5G07990.1**	1542	AT5G07990.1	1542	100.0	Perfect RBH
F3H	Artha	**AT3G51240.1**	1077	AT3G51240.1	1077	100.0	Perfect RBH
ANS	Caann	**Caann_XM_016712446.1**	1287	Cannu_CA01g03670	1287	99.5	Imperfect RBH
CHI	Caann	**Caann_XM_016697793.1**	699	Cannu_CA11g02280	612	99.7	Imperfect RBH
CHS	Caann	-	-	**Cannu_CA00g90780**	390	-	No RBH
CHS	Caann	-	-	**Cannu_CA05g17040**	741	-	No RBH
CHS	Caann	**Caann_XM_016694194.1**	1212	Cannu_CA12g20070	1011	99.6	Imperfect RBH
CHS	Caann	**Caann_XM_016695870.1**	1173	Cannu_CA00g90800	1173	100.0	Perfect RBH
CHS	Caann	**Caann_XM_016695871.1**	1170	Cannu_CA00g90790	984	99.8	Imperfect RBH
CHS	Caann	**Caann_XM_016696552.1**	1158	Cannu_CA12g20050 + Cannu_CA12g20060	1053	88.4	cluster BH
CHS	Caann	**Caann_XM_016710598.1**	1170	Cannu_CA03g02050	1170	99.9	Imperfect RBH
CHS	Caann	**Caann_XM_016718115.1**	1179	-	-	-	No RBH
CHS	Caann	**Caann_XM_016718139.1**	1209	Cannu_CA05g17060	1209	99.8	Imperfect RBH
CHS	Caann	**Caann_XM_016726963.1**	798	Cannu_CA08g19650	798	100.0	Perfect RBH
DFR	Caann	**Caann_XM_016705224.1**	1236	Cannu_CA02g22270	1101	100.0	Imperfect RBH
F3′5′H	Caann	**Caann_XM_016691378.1**	1536	-	-	-	No RBH
F3′5′H	Caann	**Caann_XM_016693437.1**	1542	Cannu_CA11g18550	1374	99.9	Imperfect RBH
F3′H	Caann	**Caann_XM_016707872.1**	1542	Cannu_CA03g29350	1257	98.9	Imperfect RBH
F3H	Caann	-	-	**Cannu_CA02g21520**	546	-	No RBH
F3H	Caann	**Caann_XM_016705025.1**	1098	Cannu_CA02g21550	1101	99.7	Imperfect RBH
ANS	Cocan	-	-	**Ccan_Cc08_g13720**	-	-	No RBH
CHI	Cocan	-	-	**Ccan_Cc02_g08570**	-	-	No RBH
CHS	Cocan	-	-	**Ccan_Cc00_g19010**	-	-	No RBH
CHS	Cocan	-	-	**Ccan_Cc00_g32030**	-	-	No RBH
DFR	Cocan	-	-	**Ccan_Cc07_g09810**	-	-	No RBH
F3′H	Cocan	-	-	**Ccan_Cc00_g07110**	-	-	No RBH
F3′H	Cocan	-	-	**Ccan_Cc00_g07120**	-	-	No RBH
F3′H	Cocan	-	-	**Ccan_Cc02_g28860**	-	-	No RBH
F3′H	Cocan	-	-	**Ccan_Cc02_g28870**	-	-	No RBH
F3′H	Cocan	-	-	**Ccan_Cc04_g13260**	-	-	No RBH
F3H	Cocan	-	-	**Ccan_Cc07_g10740**	-	-	No RBH
ANS	Cycar	**Cycar_XM_025103808.1**	-	-	-	-	No RBH
CHI	Cycar	**Cycar_XM_025113756.1**	-	-	-	-	No RBH
CHI	Cycar	**Cycar_XM_025114537.1**	-	-	-	-	No RBH
CHS	Cycar	**Cycar_XM_025115139.1**	-	-	-	-	No RBH
CHS	Cycar	**Cycar_XM_025115340.1**	-	-	-	-	No RBH
CHS	Cycar	**Cycar_XM_025117980.1**	-	-	-	-	No RBH
CHS	Cycar	**Cycar_XM_025124791.1**	-	-	-	-	No RBH
CHS	Cycar	**Cycar_XM_025126676.1**	-	-	-	-	No RBH
CHS	Cycar	**Cycar_XM_025126677.1**	-	-	-	-	No RBH
DFR	Cycar	**Cycar_XM_025113289.1**	-	-	-	-	No RBH
F3′H	Cycar	**Cycar_XM_025120904.1**	-		-	-	No RBH
F3H	Cycar	**Cycar_XM_025118318.1**	-		-	-	No RBH
ANS	Dacar	**Dacar_XM_017380613.1**	1209	DCAR_006772	1209	100.0	Perfect RBH
CHI	Dacar	**Dacar_XM_017365109.1**	879	DCAR_027694	663	100.0	Imperfect RBH
CHS	Dacar	**Dacar_XM_017360477.1**	1176	DCAR_026111	1176	100.0	Perfect RBH
CHS	Dacar	**Dacar_XM_017364131.1**	1176	DCAR_026110	921	99.4	cluster BH
CHS	Dacar	**Dacar_XM_017368840.1**	1170	DCAR_030786	1170	100.0	Perfect RBH
CHS	Dacar	**Dacar_XM_017371435.1**	1173	DCAR_030785	762	100.0	Imperfect RBH
CHS	Dacar	**Dacar_XM_017372560.1**	453	DCAR_000081	465	100.0	Imperfect RBH
CHS	Dacar	**Dacar_XM_017384350.1**	753	-	-	-	No RBH

CHS	Dacar	**Dacar_XM_017388617.1**	1176	DCAR_014462	774	100.0	Imperfect RBH
CHS	Dacar	**Dacar_XM_017394258.1**	1179	DCAR_018997	1179	100.0	Perfect RBH
CHS	Dacar	**Dacar_XM_017395620.1**	495	-	-	-	No RBH
CHS	Dacar	**Dacar_XM_017396858.1**	1233	DCAR_018999	1197	100.0	cluster BH
CHS	Dacar	**Dacar_XM_017396859.1**	1233	DCAR_019001	1197	100.0	Imperfect RBH
CHS	Dacar	**Dacar_XM_017396860.1**	1179	DCAR_019002	1179	100.0	Imperfect RBH
CHS	Dacar	**Dacar_XM_017397154.1**	1194	DCAR_018992	1128	100.0	cluster BH
DFR	Dacar	**Dacar_XM_017389661.1**	1014	DCAR_013832	1014	100.0	Perfect RBH
DFR	Dacar	**Dacar_XM_017394438.1**	1008	DCAR_017533	813	97.7	cluster BH
DFR	Dacar	**Dacar_XM_017395352.1 split1**	**1005**	DCAR_017529	1005	100.0	Imperfect RBH
DFR	Dacar	**Dacar_XM_017395352.1 split2**	**1005**	DCAR_017530	1005	98.3	Imperfect RBH
DFR	Dacar	**Dacar_XM_017396019.1**	1014	DCAR_017528	1008	100.0	Imperfect RBH
DFR	Dacar	**Dacar_XM_017396664.1**	1044	-	-	-	No RBH
DFR	Dacar	**Dacar_XM_017396665.1**	1002	DCAR_017531	1014	97.5	Imperfect RBH
DFR	Dacar	**Dacar_XM_017398898.1**	1056	DCAR_022969	1056	100.0	Perfect RBH
DFR	Dacar	**Dacar_XM_017399501.1**	1143	DCAR_021485	1140	99.7	Imperfect RBH
F3′H	Dacar	**Dacar_XM_017388905.1**	1551	DCAR_014032	1356	100.0	Imperfect RBH
F3H	Dacar	**Dacar_XM_017383706.1**	936	DCAR_009487	699	100.0	Imperfect RBH
F3H	Dacar	**Dacar_XM_017385170.1**	1149	DCAR_009488	711	100.0	Imperfect RBH
F3H	Dacar	**Dacar_XM_017385173.1**	1101	DCAR_009483	828	100.0	Imperfect RBH
F3H	Dacar	**Dacar_XM_017385175.1**	1074	DCAR_009489	726	100.0	Imperfect RBH
ANS	Heann	**Heann_XM_022177923.1**	1083	HanXRQChr04g0095451	1134	100.0	Imperfect RBH
ANS	Heann	**Heann_XM_022177924.1**	1068	HanXRQChr04g0095471	1068	100.0	Perfect RBH
CHI	Heann	**Heann_XM_022155533.1**	684	HanXRQChr15g0496901	669	100.0	Imperfect RBH
CHI	Heann	**Heann_XM_022157901.1**	672	HanXRQChr15g0496871	672	100.0	Perfect RBH
CHI	Heann	**Heann_XM_022157902.1**	666	HanXRQChr15g0496881	666	100.0	Perfect RBH
CHS	Heann	**Heann_XM_022127208.1**	1227	HanXRQChr09g0260921	1227	100.0	Perfect RBH
CHS	Heann	**Heann_XM_022127919.1**	1365	HanXRQChr09g0267961	1386	100.0	Imperfect RBH
CHS	Heann	**Heann_XM_022136927.1**	1212	HanXRQChr11g0324891	1212	100.0	Perfect RBH
CHS	Heann	**Heann_XM_022136928.1**	1299	HanXRQChr11g0324921	1239	100.0	Imperfect RBH
CHS	Heann	**Heann_XM_022136929.1**	1224	HanXRQChr11g0324941	960	100.0	Imperfect RBH
CHS	Heann	**Heann_XM_022136930.1**	609	HanXRQChr11g0324911	579	100.0	Imperfect RBH
CHS	Heann	**Heann_XM_022142672.1**	1209	HanXRQChr12g0375361	1209	100.0	Perfect RBH
CHS	Heann	**Heann_XM_022153962.1**	1197	HanXRQChr14g0440901	1344	100.0	Imperfect RBH
CHS	Heann	**Heann_XM_022153975.1**	1197	HanXRQChr14g0441041	1197	100.0	Perfect RBH
CHS	Heann	**Heann_XM_022161268.1**	1236	HanXRQChr16g0508841	1335	100.0	Imperfect RBH
CHS	Heann	**Heann_XM_022178725.1**	1197	HanXRQChr04g0106111	1197	100.0	Perfect RBH
CHS	Heann	**Heann_XM_022179491.1**	1170	HanXRQChr04g0114951	1254	100.0	cluster BH
CHS	Heann	**Heann_XM_022179492.1**	1170	HanXRQChr04g0114941	1170	100.0	Perfect RBH
DFR	Heann	**Heann_XM_022145746.1**	1068	HanXRQChr13g0407201	1068	100.0	Perfect RBH
F3′H	Heann	**Heann_XM_022119664.1**	705	HanXRQChr08g0233711	543	100.0	Imperfect RBH
F3′H	Heann	**Heann_XM_022122577.1**	1539	HanXRQChr08g0233771	1617	100.0	Imperfect RBH
F3′H	Heann	**Heann_XM_022124823.1**	504	HanXRQChr09g0266651	423	100.0	Imperfect RBH
F3′H	Heann	**Heann_XM_022153126.1**	1518	HanXRQChr14g0430831	1518	100.0	Perfect RBH
F3′H	Heann	**Heann_XM_022153244.1**	1539	HanXRQChr14g0432191	1539	100.0	Perfect RBH
F3H	Heann	**Heann_XM_022160179.1**	1095	HanXRQChr16g0531451	1095	100.0	Perfect RBH
ANS	Ipnil	**Ipnil_XM_019317185.1**	1116	INIL13g40254.t1	1116	100.0	Perfect RBH
CHI	Ipnil	**Ipnil_XM_019337142.1**	726	INIL11g18443.t1	726	100.0	Perfect RBH
CHS	Ipnil	-	-	**INIL11g18930.t1**	1191	-	No RBH
CHS	Ipnil	**Ipnil_XM_019310063.1**	1170	INIL14g35461.t1	1170	100.0	Perfect RBH
CHS	Ipnil	**Ipnil_XM_019326011.1**	1167	INIL12g08537.t1	1167	100.0	Perfect RBH
CHS	Ipnil	**Ipnil_XM_019339953.1**	1191	INIL11g18928.t1	1191	100.0	Perfect RBH
CHS	Ipnil	**Ipnil_XM_019339957.1**	1182	INIL11g18931.t1	1182	100.0	Perfect RBH
CHS	Ipnil	**Ipnil_XM_019339958.1**	1134	INIL11g18926.t1	1134	100.0	Perfect RBH
DFR	Ipnil	**Ipnil_XM_019327016.1**	1074	INIL05g09558.t1	1044	100.0	Imperfect RBH
DFR	Ipnil	**Ipnil_XM_019327030.1**	1212	INIL05g09559.t1	1212	100.0	Perfect RBH
DFR	Ipnil	**Ipnil_XM_019327031.1**	1071	INIL05g09560.t1	1071	100.0	Perfect RBH
F3′H	Ipnil	**Ipnil_XM_019308867.1**	1560	INIL04g34064.t1	756	100.0	Imperfect RBH
F3H	Ipnil	**Ipnil_XM_019316832.1**	1101	INIL02g39928.t1	1077	97.8	Imperfect RBH

F3H	Ipnil	**Ipnil_XM_019316833.1**	1002	INIL02g39929.t1	1002	100.0	Perfect RBH
F3H	Ipnil	**Ipnil_XM_019327227.1**	1086	INIL05g09512.t1	1086	100.0	Perfect RBH
ANS	Lasat	-	-	**Lsat_1_v5_gn_9_97280.1**	681	-	No RBH
CHI	Lasat	**Lasat_XM_023891334.1**	708	Lsat_1_v5_gn_9_66221.1	708	100.0	Perfect RBH
CHS	Lasat	-	-	**Lsat_1_v5_gn_2_42901.1**	531	-	No RBH
CHS	Lasat	**Lasat_XM_023878437.1**	1170	Lsat_1_v5_gn_2_42860.1	1170	100.0	Perfect RBH
CHS	Lasat	**Lasat_XM_023878440.1**	1170	Lsat_1_v5_gn_2_43721.1	1161	100.0	Imperfect RBH
CHS	Lasat	**Lasat_XM_023878441.1**	1170	-	-	-	No RBH
CHS	Lasat	**Lasat_XM_023878447.1**	1176	Lsat_1_v5_gn_2_42881.1	1170	100.0	Imperfect RBH
CHS	Lasat	**Lasat_XM_023878452.1**	1200	Lsat_1_v5_gn_2_43780.1	1170	100.0	Imperfect RBH
CHS	Lasat	**Lasat_XM_023878453.1**	1152	Lsat_1_v5_gn_2_43681.1	1152	100.0	Perfect RBH
CHS	Lasat	**Lasat_XM_023878454.1**	1185	Lsat_1_v5_gn_2_42941.1	1170	100.0	Imperfect RBH
CHS	Lasat	**Lasat_XM_023879789.1**	1200	Lsat_1_v5_gn_2_76880.1	1197	100.0	Imperfect RBH
CHS	Lasat	**Lasat_XM_023897706.1**	1215	Lsat_1_v5_gn_4_147220.1	1215	100.0	Perfect RBH
DFR	Lasat	**Lasat_XM_023893302.1**	1062	Lsat_1_v5_gn_2_77261.1	1062	100.0	Perfect RBH
F3′H	Lasat	**Lasat_XM_023887166.1**	1539	Lsat_1_v5_gn_5_23101.1	1539	100.0	Perfect RBH
F3′H	Lasat	**Lasat_XM_023904089.1**	1557	Lsat_1_v5_gn_5_22780.1	1551	100.0	Imperfect RBH
F3H	Lasat	**Lasat_XM_023893557.1**	1095	Lsat_1_v5_gn_3_74560.1	1095	100.0	Perfect RBH
ANS	Metru	**Metru_XM_003611141.2**	1071	Medtr5g011250.1	1071	100.0	Perfect RBH
CHI	Metru	**Metru_XM_003592713.2**	669	Medtr1g115820.1	669	100.0	Perfect RBH
CHI	Metru	**Metru_XM_003592715.2**	678	Medtr1g115840.1	678	100.0	Perfect RBH
CHI	Metru	**Metru_XM_003592718.2**	675	Medtr1g115870.1	675	100.0	Perfect RBH
CHI	Metru	**Metru_XM_003592720.2**	681	Medtr1g115890.1	681	100.0	Perfect RBH
CHI	Metru	**Metru_XM_013592455.1**	465	Medtr7g027135.1	465	100.0	Perfect RBH
CHI	Metru	**Metru_XM_013615033.1**	693	Medtr1g115850.2	693	100.0	Perfect RBH
CHS	Metru	**Metru_XM_003591974.2**	1269	Medtr1g097910.1	1170	100.0	Imperfect RBH
CHS	Metru	**Metru_XM_003591996.2**	1170	Medtr1g098140.1	1170	100.0	Perfect RBH
CHS	Metru	**Metru_XM_003595597.2**	1170	Medtr2g058470.1	1170	100.0	Perfect RBH
CHS	Metru	**Metru_XM_003601599.2**	1170	Medtr3g083910.1	1170	100.0	Perfect RBH
CHS	Metru	**Metru_XM_003601600.2**	1170	Medtr3g083920.1	1170	100.0	Perfect RBH
CHS	Metru	**Metru_XM_003601819.2**	1170	Medtr3g086260.1	1170	100.0	Perfect RBH
CHS	Metru	**Metru_XM_003610804.2**	1170	Medtr5g007730.1	1170	100.0	Perfect RBH
CHS	Metru	**Metru_XM_003610805.2**	981	Medtr5g007740.1	981	100.0	Perfect RBH
CHS	Metru	**Metru_XM_003610807.2**	1170	Medtr5g007760.1	1170	100.0	Perfect RBH
CHS	Metru	**Metru_XM_003610808.1**	810	Medtr5g007770.1	810	100.0	Perfect RBH
CHS	Metru	**Metru_XM_003621482.2**	1170	Medtr7g016700.1	1170	100.0	Perfect RBH
CHS	Metru	**Metru_XM_003621484.2**	1170	Medtr7g016720.1	1170	100.0	Perfect RBH
CHS	Metru	**Metru_XM_003621486.1**	1170	Medtr7g016780.1	1170	100.0	Perfect RBH
CHS	Metru	**Metru_XM_003621488.1**	1170	Medtr7g016800.1	1170	100.0	Perfect RBH
CHS	Metru	**Metru_XM_003621490.2**	1170	Medtr7g016820.1	1170	100.0	Perfect RBH
CHS	Metru	**Metru_XM_003624476.2**	1176	Medtr7g084300.1	1176	100.0	Perfect RBH
CHS	Metru	**Metru_XM_013595036.1**	912	Medtr7g113405.1	912	100.0	Perfect RBH
CHS	Metru	**Metru_XM_013597892.1**	1242	Medtr5g007713.1	1170	100.0	Imperfect RBH
CHS	Metru	**Metru_XM_013597893.1**	1170	Medtr5g007717.1	1170	100.0	Perfect RBH
CHS	Metru	**Metru_XM_013597894.1**	1197	Medtr5g007723.1	1197	100.0	Perfect RBH
CHS	Metru	**Metru_XM_013605871.1**	1230	Medtr3g088670.1	1179	100.0	Imperfect RBH
CHS	Metru	**Metru_XM_013605872.1**	1182	Medtr3g088675.1	1182	100.0	Perfect RBH
CHS	Metru	**Metru_XM_013614150.1**	1272	Medtr1g097935.1	1170	100.0	Imperfect RBH
DFR	Metru	**Metru_XM_013610679.1**	1020	Medtr1g022440.1	1020	100.0	Perfect RBH
DFR	Metru	**Metru_XM_013610680.1**	1005	Medtr1g022445.1	1005	100.0	Perfect RBH
F3′5′H	Metru	**Metru_XM_013603872.1**	1548	Medtr3g436390.1	1521	100.0	Imperfect RBH
F3′5′H	Metru	**Metru_XM_013603876.1**	1548	Medtr3g436540.1	1548	100.0	Perfect RBH
F3′H	Metru	**Metru_XM_003598897.2**	1551	Medtr3g025230.1	1551	100.0	Perfect RBH
F3′H	Metru	**Metru_XM_003598899.1**	861	Medtr3g025250.1	861	100.0	Perfect RBH
F3′H	Metru	**Metru_XM_003598900.2**	1551	Medtr3g025260.1	1551	100.0	Perfect RBH
F3′H	Metru	**Metru_XM_013602525.1**	1533	Medtr4g109470.1	1533	100.0	Perfect RBH
F3′H	Metru	**Metru_XM_013603646.1**	1551	Medtr3g024520.1	1551	100.0	Perfect RBH
F3H	Metru	**Metru_XM_003612118.2**	1074	Medtr5g022020.1	1074	100.0	Perfect RBH
F3H	Metru	**Metru_XM_003629272.2**	1098	Medtr8g075830.1	1098	100.0	Perfect RBH
F3H	Metru	**Metru_XM_003629275.2**	1098	Medtr8g075890.1	1098	100.0	Perfect RBH
F3H	Metru	**Metru_XM_013604559.1**	1065	Medtr3g058610.1	1065	100.0	Perfect RBH

ANS	Migut	**Migut_XM_012991981.1**	1161	Migut.E01023.1	1182	100.0	Imperfect RBH
CHI	Migut	**Migut_XM_012978000.1**	756	Migut.D00159.1	720	100.0	Imperfect RBH
CHI	Migut	**Migut_XM_012982352.1**	531	-	-	-	No RBH
CHS	Migut	**Migut_XM_012973820.1**	1179	Migut.G00627.1	1179	100.0	Perfect RBH
CHS	Migut	**Migut_XM_012973831.1**	1173	Migut.G00628.1	1173	100.0	Perfect RBH
CHS	Migut	**Migut_XM_012982223.1**	501	Migut.B01017.1	504	100.0	Imperfect RBH
CHS	Migut	**Migut_XM_012988049.1**	501	Migut.H00487.1	579	100.0	cluster BH
CHS	Migut	**Migut_XM_012988726.1**	1164	Migut.G00629.1	1080	100.0	Imperfect RBH
CHS	Migut	**Migut_XM_012988738.1**	843	Migut.G00630.1	597	100.0	Imperfect RBH
DFR	Migut	**Migut_XM_012989820.1**	1230	Migut.H02531.1	1296	99.6	Imperfect RBH
F3′H	Migut	**Migut_XM_012982675.1**	1107	Migut.O00721.1	1032	100.0	Imperfect RBH
F3′H	Migut	**Migut_XM_012998441.1**	1617	Migut.D01697.1	1617	100.0	Perfect RBH
F3H	Migut	**Migut_XM_012980274.1**	1128	Migut.C00836.1	1128	100.0	Perfect RBH
F3H	Migut	**Migut_XM_012983725.1**	1071	Migut.N03161.1	1071	100.0	Perfect RBH
ANS	Nisyl	**Nisyl_XM_009777817.1**	1260	Nsyl_mRNA_82018_cds	1260	100.0	Perfect RBH
CHI	Nisyl	**Nisyl_XM_009805808.1**	735	Nsyl_mRNA_10796_cds	735	100.0	Perfect RBH
CHS	Nisyl	**Nisyl_XM_009766636.1**	1170	Nsyl_mRNA_65311_cds	1170	100.0	Perfect RBH
CHS	Nisyl	**Nisyl_XM_009768850.1**	1296	Nsyl_mRNA_68684_cds	1296	100.0	Perfect RBH
CHS	Nisyl	**Nisyl_XM_009776625.1**	1170	Nsyl_mRNA_80210_cds	1170	100.0	Perfect RBH
CHS	Nisyl	**Nisyl_XM_009789215.1**	1173	Nsyl_mRNA_25875_cds	1173	100.0	Perfect RBH
DFR	Nisyl	-	-	**Nsyl_mRNA_14898_cds**	468	-	No RBH
DFR	Nisyl	**Nisyl_XM_009761654.1**	1146	Nsyl_mRNA_57456_cds	1146	100.0	Perfect RBH
F3′5′H	Nisyl	**Nisyl_XM_009790406.1**	993	Nsyl_mRNA_27603_cds	639	100.0	Imperfect RBH
F3′H	Nisyl	**Nisyl_XM_009773108.1**	1548	Nsyl_mRNA_74969_cds	1056	99.9	cluster BH
F3′H	Nisyl	**Nisyl_XM_009790192.1**	1545	Nsyl_mRNA_27286_cds	1641	100.0	Imperfect RBH
F3H	Nisyl	**Nisyl_XM_009767241.1**	1110	Nsyl_mRNA_66230_cds	1110	100.0	Perfect RBH
ANS	Nitab	**Nitab_XM_016598316.1**	1260	Nitab4.5_0010547g0010.1	1125	100.0	Imperfect RBH
ANS	Nitab	**Nitab_XM_016645479.1**	1260	Nitab4.5_0003274g0110.1	1098	100.0	Imperfect RBH
CHI	Nitab	**Nitab_XM_016600175.1**	735	Nitab4.5_0005313g0010.1	645	100.0	cluster BH
CHI	Nitab	**Nitab_XM_016652447.1**	735	Nitab4.5_0000027g0470.1	735	100.0	Perfect RBH
CHS	Nitab	-	-	**Nitab4.5_0001998g0020.1**	1023	-	No RBH
CHS	Nitab	-	-	**Nitab4.5_0003185g0100.1 split2**	1066	-	No RBH
CHS	Nitab	**Nitab_XM_016624553.1**	1296	Nitab4.5_0010357g0030.1	1233	97.9	Imperfect RBH
CHS	Nitab	**Nitab_XM_016625162.1**	1170	Nitab4.5_0003904g0060.1	1107	100.0	Imperfect RBH
CHS	Nitab	**Nitab_XM_016634418.1**	1170	Nitab4.5_0001066g0070.1	1170	99.9	Imperfect RBH
CHS	Nitab	**Nitab_XM_016638700.1**	1173	Nitab4.5_0003185g0100.1 split1	1101	93.2	Imperfect RBH
CHS	Nitab	**Nitab_XM_016638898.1**	1170	Nitab4.5_0002920g0060.1	762	100.0	Imperfect RBH
CHS	Nitab	**Nitab_XM_016653399.1**	1170	Nitab4.5_0014110g0010.1	1326	100.0	Imperfect RBH
CHS	Nitab	**Nitab_XM_016660053.1**	1173	Nitab4.5_0006945g0020.1	1107	100.0	Imperfect RBH
DFR	Nitab	**Nitab_XM_016620104.1**	1146	Nitab4.5_0007490g0080.1	1014	100.0	Imperfect RBH
DFR	Nitab	**Nitab_XM_016622310.1**	399	Nitab4.5_0007770g0040.1	816	100.0	cluster BH
DFR	Nitab	**Nitab_XM_016626713.1**	1149	Nitab4.5_0000178g0360.1	1053	100.0	Imperfect RBH
F3′5′H	Nitab	**Nitab_XM_016597113.1**	1590	Nitab4.5_0000173g0310.1	1524	100.0	Imperfect RBH
F3′H	Nitab	**Nitab_XM_016592903.1**	1548	Nitab4.5_0001410g0070.1	1641	99.2	Imperfect RBH
F3′H	Nitab	**Nitab_XM_016618350.1**	1548	Nitab4.5_0001485g0210.1	1587	99.9	cluster BH
F3′H	Nitab	**Nitab_XM_016656982.1**	591	Nitab4.5_0001410g0020.1	1419	100.0	cluster BH
F3H	Nitab	**Nitab_XM_016590223.1**	1110	Nitab4.5_0000864g0050.1	1110	100.0	Perfect RBH
F3H	Nitab	**Nitab_XM_016630614.1**	1110	Nitab4.5_0026775g0010.1	1110	100.0	Perfect RBH
ANS	Nitom	**Nitom_XM_009605813.2**	1260	Ntom_mRNA_13259_cds	1260	100.0	Perfect RBH
CHI	Nitom	**Nitom_XM_009588739.2**	735	Ntom_mRNA_7985_cds	735	100.0	Perfect RBH
CHS	Nitom	**Nitom_NM_001319862.1**	1170	Ntom_mRNA_18483_cds	1170	100.0	Perfect RBH
CHS	Nitom	**Nitom_XM_009610476.2**	1173	Ntom_mRNA_27571_cds	1173	100.0	Perfect RBH
CHS	Nitom	**Nitom_XM_009610477.2**	1170	Ntom_mRNA_27574_cds	1170	100.0	Perfect RBH
CHS	Nitom	**Nitom_XM_009618342.2**	1170	Ntom_mRNA_38569_cds	1170	100.0	Perfect RBH
DFR	Nitom	**Nitom_NM_001302522.1**	1149	Ntom_mRNA_5184_cds	1149	99.8	Imperfect RBH
F3′5′H	Nitom	**Nitom_XM_009621551.2**	1524	Ntom_mRNA_18488_cds	1524	100.0	Perfect RBH
F3′H	Nitom	**Nitom_XM_009598972.2**	1548	Ntom_mRNA_74743_cds	1548	100.0	Perfect RBH
F3H	Nitom	**Nitom_XM_009605261.2**	1110	Ntom_mRNA_83308_cds	1110	100.0	Perfect RBH
ANS	Oleur	**Oleur_XM_022990294.1**	1062	Oeu059047.1	1014	100.0	Imperfect RBH
CHI	Oleur	**Oleur_KF886191.1**	750	Oeu057578.1	381	100.0	Imperfect RBH

CHS	Oleur	**Oleur_XM_022997057.1**	1173	Oeu006719.1	1173	100.0	Perfect RBH
CHS	Oleur	**Oleur_XM_022997058.1**	1173	Oeu006720.1	1173	100.0	Perfect RBH
CHS	Oleur	**Oleur_XM_023002817.1**	1173	Oeu022348.1	1173	100.0	Perfect RBH
CHS	Oleur	**Oleur_XM_023018865.1**	1170	Oeu016712.1	1170	100.0	Perfect RBH
DFR	Oleur	**Oleur_XM_022999201.1**	351	Oeu012314.1	351	100.0	Perfect RBH
DFR	Oleur	**Oleur_XM_023039626.1**	759	Oeu057948.1	561	100.0	Imperfect RBH
F3′H	Oleur	**Oleur_XM_023029392.1**	1644	-	-	-	No RBH
F3H	Oleur	**Oleur_XM_023003872.1**	387	-	-	-	No RBH
F3H	Oleur	**Oleur_XM_023028959.1**	1110	Oeu046264.1	1110	100.0	Perfect RBH
ANS	Peaxi	-	-	**Peaxi162Scf00620g00533.1**	-	-	No RBH
CHI	Peaxi	-	-	**Peaxi162Scf00006g00088.1**	-	-	No RBH
CHI	Peaxi	-	-	**Peaxi162Scf00038g01957.1**	-	-	No RBH
CHS	Peaxi	-	-	**Peaxi162Scf00047g01225.1**	-	-	No RBH
CHS	Peaxi	-	-	**Peaxi162Scf00126g01326.1**	-	-	No RBH
CHS	Peaxi	-	-	**Peaxi162Scf00164g00313.1**	-	-	No RBH
CHS	Peaxi	-	-	**Peaxi162Scf00536g00092.1**	-	-	No RBH
CHS	Peaxi	-	-	**Peaxi162Scf00817g00216.1**	-	-	No RBH
CHS	Peaxi	-	-	**Peaxi162Scf00830g00016.1**	-	-	No RBH
CHS	Peaxi	-	-	**Peaxi162Scf00830g00048.1**	-	-	No RBH
CHS	Peaxi	-	-	**Peaxi162Scf00830g00067.1**	-	-	No RBH
CHS	Peaxi	-	-	**Peaxi162Scf00938g00001.1**	-	-	No RBH
CHS	Peaxi	-	-	**Peaxi162Scf00938g00118.1**	-	-	No RBH
CHS	Peaxi	-	-	**Peaxi162Scf08277g00001.1**	-	-	No RBH
DFR	Peaxi	-	-	**Peaxi162Scf00366g00630.1**	-	-	No RBH
F3′5′H	Peaxi	-	-	**Peaxi162Scf00108g00417.1**	-	-	No RBH
F3′5′H	Peaxi	-	-	**Peaxi162Scf00150g00218.1**	-	-	No RBH
F3′H	Peaxi	-	-	**Peaxi162Scf00201g00243.1**	-	-	No RBH
F3H	Peaxi	-	-	**Peaxi162Scf00328g01214.1**	-	-	No RBH
ANS	Potri	**Potri_XM_002298045.2**	1083	Potri.001G113100.1	1083	100.0	Perfect RBH
ANS	Potri	**Potri_XM_002304416.3**	1197	Potri.003G119100.1	1086	100.0	Imperfect RBH
CHI	Potri	**Potri_XM_002315222.3**	672	Potri.010G213000.1	675	99.6	Imperfect RBH
CHS	Potri	**Potri_XM_002299161.3**	1131	Potri.001G028600.1	1131	100.0	Perfect RBH
CHS	Potri	**Potri_XM_002299245.3**	1176	Potri.001G051500.1	1176	100.0	Perfect RBH
CHS	Potri	**Potri_XM_002299246.3**	1173	Potri.001G051600.1	1173	100.0	Perfect RBH
CHS	Potri	**Potri_XM_002303783.3**	1176	Potri.003G176700.1	1176	100.0	Perfect RBH
CHS	Potri	**Potri_XM_002303784.3**	1176	Potri.003G176800.1	1176	100.0	Perfect RBH
CHS	Potri	**Potri_XM_002303785.3**	1176	Potri.003G176900.1	1176	100.0	Perfect RBH
CHS	Potri	**Potri_XM_002321045.3**	1191	Potri.014G145100.1	1206	98.8	Imperfect RBH
CHS	Potri	**Potri_XM_006377012.2**	1188	Potri.012G138800.1	1188	100.0	Perfect RBH
CHS	Potri	**Potri_XM_006383408.2**	1182	Potri.005G175200.1	1014	100.0	Imperfect RBH
DFR	Potri	**Potri_XM_002300723.3**	1041	Potri.002G033600.1	1041	100.0	Perfect RBH
DFR	Potri	**Potri_XM_006383649.2**	1131	Potri.005G229500.1	1164	100.0	Imperfect RBH
F3′5′H	Potri	**Potri_XM_002298428.2**	1527	Potri.001G274600.1	1527	100.0	Perfect RBH
F3′5′H	Potri	**Potri_XM_002313968.3**	1530	Potri.009G069100.1	1530	100.0	Perfect RBH
F3′H	Potri	**Potri_XM_002319725.3**	1566	Potri.013G073300.1	1566	100.0	Perfect RBH
F3H	Potri	-	-	**Potri.005G114000.1**	1158	-	No RBH
F3H	Potri	**Potri_XM_006383041.2**	1092	Potri.005G113700.1	1092	100.0	Perfect RBH
F3H	Potri	**Potri_XM_006383044.2**	1092	Potri.005G113900.1	1092	100.0	Perfect RBH
ANS	Seind	**Seind_XM_011073005.2**	1092	SIN_1026305	1092	100.0	Perfect RBH
CHI	Seind	**Seind_XM_011081420.2**	669	-	-	-	No RBH
CHS	Seind	**Seind_XM_011073310.2**	1161	-	-	-	No RBH
CHS	Seind	**Seind_XM_011093097.2**	1176	SIN_1018959	1176	100.0	Perfect RBH
CHS	Seind	**Seind_XM_011093098.2**	1176	SIN_1018960	1176	100.0	Perfect RBH
CHS	Seind	**Seind_XM_011093100.2**	1173	SIN_1018961	1173	100.0	Perfect RBH
DFR	Seind	**Seind_XM_011084901.2**	1146	SIN_1022200	1047	99.9	Imperfect RBH
F3′H	Seind	**Seind_XM_011074564.2**	1593	SIN_1017435	1536	100.0	Imperfect RBH
F3H	Seind	**Seind_XM_011085253.2**	1101	SIN_1002345	1101	100.0	Perfect RBH
ANS	Solyc	**Solyc_XM_010327136.3**	1326	Solyc08g080040.3.1	1311	100.0	Imperfect RBH
CHI	Solyc	**Solyc_NM_001320711.1**	681	Solyc05g010320.3.1	615	100.0	Imperfect RBH
CHI	Solyc	**Solyc_XM_004239382.4**	669	Solyc05g010310.3.1	669	100.0	Perfect RBH
CHS	Solyc	**Solyc_NM_001247104.2**	1170	Solyc09g091510.3.1	1170	100.0	Perfect RBH

CHS	Solyc	**Solyc_NM_001247107.2**	1170	Solyc05g053550.3.1	1170	100.0	Perfect RBH
CHS	Solyc	**Solyc_XM_004239850.1**	1179	Solyc05g053170.3.1	1179	100.0	Perfect RBH
CHS	Solyc	**Solyc_XM_010316555.2**	1227	Solyc12g098090.1.1	1317	100.0	Imperfect RBH
CHS	Solyc	**Solyc_XM_026031338.1**	1164	Solyc06g043120.1.1 + Solyc06g043130.1.1	996	100.0	Imperfect RBH
DFR	Solyc	**Solyc_XM_010317577.3**	1149	Solyc02g085020.3.1	1149	100.0	Perfect RBH
F3′5′H	Solyc	**Solyc_NM_001247911.2**	1599	Solyc11g066580.2.1	1536	100.0	Imperfect RBH
F3′H	Solyc	**Solyc_NM_001302915.1**	1545	Solyc03g115220.3.1	1545	99.9	Imperfect RBH
F3H	Solyc	**Solyc_NM_001329483.1**	1089	Solyc02g083860.3.1	1089	100.0	Perfect RBH
ANS	Sopen	**Sopen_XM_015229000.1**	1368	Sopen08g028390.1	1368	100.0	Perfect RBH
CHI	Sopen	**Sopen_XM_015219774.1**	669	Sopen05g006080.1	669	100.0	Perfect RBH
CHI	Sopen	**Sopen_XM_015221071.1**	681	Sopen05g006090.1	603	100.0	Imperfect RBH
CHS	Sopen	-	-	**Sopen06g013590.1**	486	-	No RBH
CHS	Sopen	-	-	**Sopen12g032910.1**	1086	-	No RBH
CHS	Sopen	**Sopen_XM_015205152.1**	1191	Sopen12g032900.1	1173	100.0	Imperfect RBH
CHS	Sopen	**Sopen_XM_015221148.1**	1170	Sopen05g032070.1	1170	100.0	Perfect RBH
CHS	Sopen	**Sopen_XM_015221406.1**	1296	Sopen05g031680.1	981	100.0	Imperfect RBH
CHS	Sopen	**Sopen_XM_015232996.1**	1170	Sopen09g034440.1	1170	100.0	Perfect RBH
DFR	Sopen	**Sopen_XM_015210279.1**	1149	Sopen02g029720.1	1149	100.0	Perfect RBH
F3′5′H	Sopen	**Sopen_XM_015203537.1**	1536	Sopen11g026310.1	1368	100.0	Imperfect RBH
F3′H	Sopen	**Sopen_XM_015215550.1**	1560	Sopen03g034260.1	1368	100.0	Imperfect RBH
F3H	Sopen	**Sopen_XM_015211594.1**	1089	Sopen02g028650.1	1089	100.0	Perfect RBH
ANS	Sotub	**Sotub_XM_006358783.2**	1368	PGSC0003DMT400058554	1368	100.0	Perfect RBH
CHI	Sotub	**Sotub_XM_006348548.2**	669	-	-	-	No RBH
CHI	Sotub	**Sotub_XM_006348549.2**	765	-	-	-	No RBH
CHS	Sotub	-	-	**PGSC0003DMT400022255**	1002	-	No RBH
CHS	Sotub	-	-	**PGSC0003DMT400022261**	438	-	No RBH
CHS	Sotub	**Sotub_NM_001288367.1**	1170	PGSC0003DMT400049165	1170	97.9	Imperfect RBH
CHS	Sotub	**Sotub_NM_001288423.1**	1170	PGSC0003DMT400076178	1170	98.3	Imperfect RBH
CHS	Sotub	**Sotub_XM_006349878.2**	1173	PGSC0003DMT400043447	1173	100.0	Perfect RBH
CHS	Sotub	**Sotub_XM_006349914.1**	1215	PGSC0003DMT400043464	1215	100.0	Perfect RBH
CHS	Sotub	**Sotub_XM_006355644.2**	1179	PGSC0003DMT400069814	1179	100.0	Perfect RBH
CHS	Sotub	**Sotub_XM_006367256.2**	1173	PGSC0003DMT400022254	1173	100.0	Perfect RBH
CHS	Sotub	**Sotub_XM_006367257.2**	1179	PGSC0003DMT400022258	1179	100.0	Perfect RBH
CHS	Sotub	**Sotub_XM_015314781.1**	1155	PGSC0003DMT400076179	1140	100.0	Imperfect RBH
DFR	Sotub	**Sotub_NM_001288480.1**	1155	PGSC0003DMT400009287	1149	100.0	Imperfect RBH
F3′5′H	Sotub	**Sotub_XM_015309422.1**	1530	PGSC0003DMT400001124	1344	100.0	Imperfect RBH
F3′H	Sotub	**Sotub_XM_006345070.2**	1545	PGSC0003DMT400063351	1368	100.0	Imperfect RBH
F3H	Sotub	**Sotub_NM_001288001.1**	1077	PGSC0003DMT400009175	1083	98.8	Imperfect RBH
ANS	Vivin	**Vivin_NM_001281218.1**	1068	GSVIVT01019892001	720	100.0	Imperfect RBH
CHI	Vivin	**Vivin_NM_001281104.1**	714	GSVIVT01032619001	705	99.4	Imperfect RBH
CHS	Vivin	**Vivin_NM_001280950.1**	1182	-	-	-	No RBH
CHS	Vivin	**Vivin_NM_001281005.1**	1179	GSVIVT01010554001	1035	99.1	cluster BH
CHS	Vivin	**Vivin_NM_001281010.1**	1179	-	-	-	No RBH
CHS	Vivin	**Vivin_NM_001281117.1**	1179	GSVIVT01010590001	1179	94.5	cluster BH
CHS	Vivin	**Vivin_NM_001281135.1**	1365	-	-	-	No RBH
CHS	Vivin	**Vivin_XM_002263686.4**	1179	GSVIVT01010556001	897	100.0	cluster BH
CHS	Vivin	**Vivin_XM_002263771.3**	1179	-	-	-	No RBH
CHS	Vivin	**Vivin_XM_002263845.4**	1179	GSVIVT01010561001	771	100.0	cluster BH
CHS	Vivin	**Vivin_XM_002263983.3**	1170	GSVIVT01000521001	300	100.0	cluster BH
CHS	Vivin	**Vivin_XM_002264419.4**	1179	GSVIVT01010565001	1095	100.0	Imperfect RBH
CHS	Vivin	**Vivin_XM_002264953.4**	1179	GSVIVT01010572001	1284	100.0	Imperfect RBH
CHS	Vivin	**Vivin_XM_002265955.3**	1161	-	-	-	No RBH
CHS	Vivin	**Vivin_XM_002268720.4**	1179	-	-	-	No RBH
CHS	Vivin	**Vivin_XM_002269350.4**	1179	-	-	-	No RBH
CHS	Vivin	**Vivin_XM_002269415.4**	1161	-	-	-	No RBH
CHS	Vivin	**Vivin_XM_002271335.4**	1179	GSVIVT01026213001	558	95.7	cluster BH
CHS	Vivin	**Vivin_XM_002272093.4**	1179	GSVIVT01026220001	729	100.0	cluster BH
CHS	Vivin	**Vivin_XM_002278318.4**	1179	GSVIVT01010589001	1179	99.9	Imperfect RBH
CHS	Vivin	**Vivin_XM_002278447.4**	1179	-	-	-	No RBH
CHS	Vivin	**Vivin_XM_003634009.3**	1155	-	-	-	No RBH
CHS	Vivin	**Vivin_XM_003634015.3**	1272	-	-	-	No RBH

CHS	Vivin	**Vivin_XM_003634016.3**	1179	GSVIVT01010563001	1053	100.0	Imperfect RBH
CHS	Vivin	**Vivin_XM_003634017.3**	1269	GSVIVT01010570001	327	91.1	cluster BH
CHS	Vivin	**Vivin_XM_003634018.3**	1275	-	-	-	No RBH
CHS	Vivin	**Vivin_XM_003634019.3**	1179	GSVIVT01010574001	990	100.0	Imperfect RBH
CHS	Vivin	**Vivin_XM_003634020.3**	1179	-	-	-	No RBH
CHS	Vivin	**Vivin_XM_003634021.3**	1179	GSVIVT01010583001	411	96.1	cluster BH
CHS	Vivin	**Vivin_XM_003634022.3**	1179	-	-	-	No RBH
CHS	Vivin	**Vivin_XM_003634023.3**	1179	GSVIVT01010579001	381	99.7	cluster BH
CHS	Vivin	**Vivin_XM_003634024.3**	1179	GSVIVT01010580001	1035	100.0	Imperfect RBH
CHS	Vivin	**Vivin_XM_003634025.3**	1179	GSVIVT01010581001	990	100.0	cluster BH
CHS	Vivin	**Vivin_XM_003634026.3**	1179	GSVIVT01010582001	1035	100.0	Imperfect RBH
CHS	Vivin	**Vivin_XM_003634027.3**	1179	GSVIVT01010584001	444	100.0	cluster BH
CHS	Vivin	**Vivin_XM_003634028.3**	1179	GSVIVT01010585001	990	100.0	cluster BH
CHS	Vivin	**Vivin_XM_010657617.2**	1101	-	-	-	No RBH
CHS	Vivin	**Vivin_XM_010664169.1**	1032	-	-	-	No RBH
CHS	Vivin	**Vivin_XM_010664197.2**	1263	GSVIVT01010568001	450	100.0	cluster BH
CHS	Vivin	**Vivin_XM_010664198.2**	1179	-	-	-	No RBH
CHS	Vivin	**Vivin_XM_010664202.2**	1179	-	-	-	No RBH
CHS	Vivin	**Vivin_XM_019224647.1**	1182	GSVIVT01032968001	1107	100.0	Imperfect RBH
CHS	Vivin	**Vivin_XM_019225976.1**	579	-	-	-	No RBH
CHS	Vivin	**Vivin_XM_019225977.1**	924	-	-	-	No RBH
DFR	Vivin	-	-	**GSVIVT01009742001**	429	-	No RBH
DFR	Vivin	**Vivin_XM_010666645.2**	1041	GSVIVT01009745001	1335	100.0	Imperfect RBH
DFR	Vivin	**Vivin_XM_019216415.1**	1020	GSVIVT01009743001	1017	100.0	Imperfect RBH
DFR	Vivin	**Vivin_XM_019219312.1**	486	GSVIVT01018927001	486	100.0	Perfect RBH
F3′5′H	Vivin	**Vivin_NM_001281228.1**	1527	-	-	-	No RBH
F3′5′H	Vivin	**Vivin_NM_001281235.1**	1527	-	-	-	No RBH
F3′5′H	Vivin	**Vivin_XM_002271703.3**	1542	GSVIVT01033633001	1659	100.0	Imperfect RBH
F3′5′H	Vivin	**Vivin_XM_002280626.3**	1530	-	-	-	No RBH
F3′5′H	Vivin	**Vivin_XM_002280903.4**	1542	-	-	-	No RBH
F3′5′H	Vivin	**Vivin_XM_003632161.3**	1542	-	-	-	No RBH
F3′5′H	Vivin	**Vivin_XM_003632164.3**	1527	-	-	-	No RBH
F3′5′H	Vivin	**Vivin_XM_003632194.3**	1542	-	-	-	No RBH
F3′5′H	Vivin	**Vivin_XM_010653221.2**	1527	-	-	-	No RBH
F3′5′H	Vivin	**Vivin_XM_010653223.2**	1527	-	-	-	No RBH
F3′5′H	Vivin	**Vivin_XM_010653225.2**	1527	-	-	-	No RBH
F3′5′H	Vivin	**Vivin_XM_010653372.1**	1215	-	-	-	No RBH
F3′5′H	Vivin	**Vivin_XM_019220452.1**	1329	-	-	-	No RBH
F3′5′H	Vivin	**Vivin_XM_019220476.1**	486	-	-	-	No RBH
F3′H	Vivin	**Vivin_NM_001280987.1**	1530	GSVIVT01007878001	1356	99.4	Imperfect RBH
F3′H	Vivin	**Vivin_XM_002284115.4**	1530	GSVIVT01007877001	588	99.5	cluster BH
F3H	Vivin	**Vivin_NM_001281105.1**	1092	GSVIVT01018781001	687	100.0	Imperfect RBH
F3H	Vivin	**Vivin_XM_002275527.3**	1077	GSVIVT01009907001	765	100.0	Imperfect RBH
F3H	Vivin	**Vivin_XM_010650732.2**	1101	GSVIVT01018780001	1320	100.0	Imperfect RBH

Annotated genes listed in bold were included in phylogenetic analyses

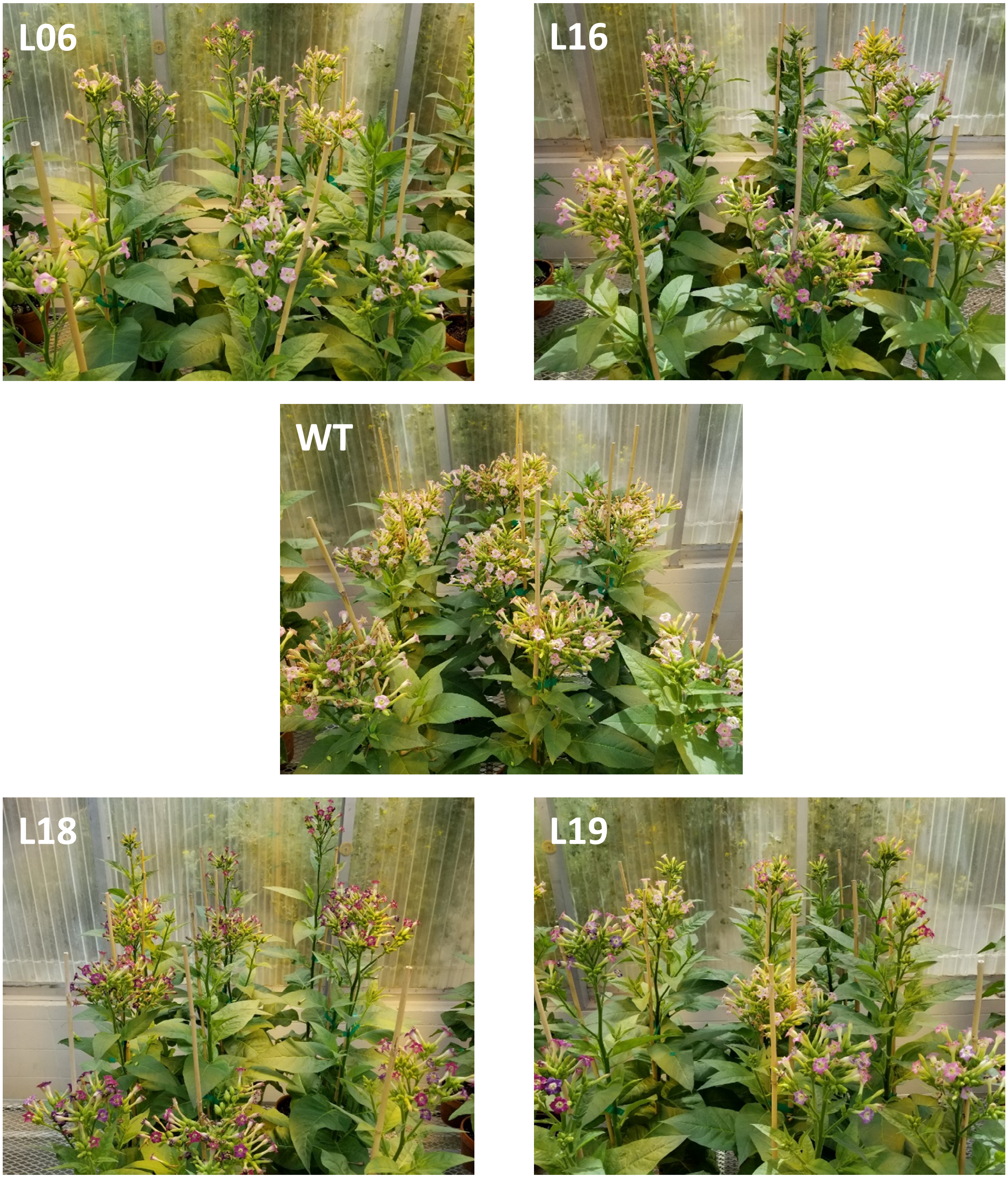

Fig S1. Populations of T_1 generation lines in comparison to WT.
